# Editorial: Surgical approaches and outcomes in cervical and thoracic myelopathies

**DOI:** 10.3389/fsurg.2025.1775565

**Published:** 2026-01-19

**Authors:** Samar S. Ayache, Georges Naïm Abi Lahoud, Moussa A. Chalah

**Affiliations:** 1 UR4391 Excitabilité Nerveuse et Thérapeutique, Faculté de Santé, Université Paris Est, Créteil, France; 2 Service de Physiologie-Explorations Fonctionnelles, DMU FIxIT, Hôpital Henri Mondor, Créteil, France; 3 Institut de la Colonne Vertébrale et des Neurosciences (ICVNS), Paris, France; 4 Department of Neurology, Gilbert and Rose-Marie Chagoury School of Medicine, Byblos, Lebanon; 5 Department of Surgery, Gilbert and Rose-Marie Chagoury School of Medicine, Byblos, Lebanon

**Keywords:** artificial intelligence, cervical myelopathy, degenerative spinal disease, laminoplasty, neurophysiology, spine surgery, surgical decompression, thoracic myelopathy

## 1 Introduction

Degenerative myelopathies are chronic, primarily non-traumatic conditions that pose a significant and growing challenge in spine care (1), accounting for an estimated 22%–50% of non-traumatic spinal cord injuries (1). The global burden of these diseases might increase with the aging population (2). These conditions can have a profound impact on patients' quality of life and impose economic and societal burdens.

Their pathophysiology is complex and multifactorial. It involves static, dynamic, as well as vascular, cellular, and molecular mechanisms (1, 3). Multiple structures could be affected including intervertebral discs, vertebral endplates, facet joints, and ligaments (1). Cervical degenerative myelopathy encompasses spondylosis-related changes, spondylolisthesis, ligamentous ossifications, and congenital, inherited, physiological, or pathological central spinal stenosis (3). Abnormal biological findings include increased neuronal ingrowth and neovascularization at the level of the degenerative discs, extracellular matrix degradation, calcification of the cartilage endplates, osteophyte formation, and dysfunction of the blood-spinal cord barrier (1).

Clinically, patients may suffer from localized pain, altered motor function (e.g., spasticity, gait disorder, altered hand dexterity) (1). These conditions may progress to ischemic injury and irreversible nerve damage, significant neurological impairment, paralysis, or even death if untreated (1). Cervical myelopathy symptoms could also include precordial pain, headaches and vertebrobasilar complaints (1). Diagnosis relies on a combination of clinical examination, neurophysiological assessment (electromyography, motor and somatosensory evoked potentials), and neuroimaging modalities (plain radiographs, magnetic resonance imaging, computed tomography) (3). However, the need for diagnostic tools capable of detecting these diseases at their earliest stages highlights an important gap and underscores the necessity for further research in this area (1).

Decompressive surgery remains the most effective management strategy for moderate to severe cases (1). Consequently, optimizing surgical approaches is a critical priority to relieve spinal cord compression, correct spinal abnormalities, enhance neurological recovery, reduce perioperative complications, and improve long-term functional outcomes in this patient population. Recent advances in spinal surgery (e.g., minimally invasive and endoscopic techniques, refinements in anterior and posterior approaches) as well as the enhanced intraoperative monitoring have substantially expanded the therapeutic armamentarium (4, 5). In parallel, emerging technologies such as machine learning algorithms are starting to be applied in surgical planning, decision-making, and risk stratification (5, 6). Intraoperative neurophysiological monitoring also plays an important role in optimizing surgical safety and outcomes (7).

In this context, this Research Topic aims to examine surgical treatments for spinal myelopathies by exploring various operative approaches and their associated outcomes. It seeks to provide surgeons and clinicians with evidence-based insights to optimize surgical techniques and enhance patient outcomes.

## 2 Overview of the published articles

The included articles collectively address a broad spectrum of clinical scenarios, surgical techniques, and perioperative challenges associated with myelopathies or related pathologies.

First, some of the articles addressed rare case reports that pose significant diagnostic and management challenges. For example, He et al. reported a case of bulbar-cervical spinal cord hemorrhage, a rare and life-threatening condition presenting with respiratory and sensorimotor symptoms. Their report underscored the critical importance of early diagnosis and prompt emergency intervention in high-level spinal cord lesions - such as microscopic hematoma evacuation, decompression via posterior cervical double-door laminectomy, and spinal stabilization - to improve prognosis and functional recovery. In a similar vein, Guo et al. described a rare case of cervical intradural disc herniation exhibiting Brown-Sequard syndrome, highlighting the diagnostic value of subtle neuroimaging findings (e.g., T2-weighted hyperintensity on magnetic resonance imaging) and intraoperative tissue characteristics (increased water content in disc tissues), as well as the importance of timely and appropriate surgical management, including cervical corpectomy and fusion with dural repair. Expanding the etiological spectrum of spinal cord compression, El Ayssami et al. highlighted the importance of early recognition and timely intervention (i.e., namely laminectomy and excision of lipomatosis) in a case of spinal epidural lipomatosis presenting with intermittent paraplegia, urinary incontinence, and sensory deficits.

Second, other articles focused on optimizing standard surgical procedures. In a retrospective study, Yang et al. evaluated posterior cervical expansive open-door laminoplasty and found no significant difference in hemostatic safety and efficacy between the use of an absorbable gelatin sponge with hemostatic fluid gelatin and tranexamic acid. Moreover, a systematic review and meta-analysis by Zheng et al. compared alternative-level vs. all-level laminoplasty and reported no differences in intraoperative outcomes or cervical sagittal parameters. However, the alternative-level procedure was associated with lower cost but also less favorable cervical canal outcomes. The generalizability of these findings is limited by the small number of available studies, all of which were conducted in a single country. Furthermore, in the context of exploring and optimizing neurophysiological monitoring during spinal surgeries, a prospective observational study by Hudec et al. found that motor evoked potentials remain reliable under deep anesthesia.

Third, other articles focused on risk stratification and complication prevention. In this context, original research by Shen et al. validated a predictive nomogram for postoperative complications following thoracic spinal stenosis in patients undergoing unilateral biportal endoscopy. This model, which incorporates clinical, radiological, and surgical variables, could support individualized perioperative planning. Additionally, Xu et al. highlighted the importance of identifying and managing procedure-related complications through a case report of postoperative myelitis following cervical spine radiofrequency ablation, underscoring the need for vigilant monitoring even in minimally invasive procedures.

## 3 Conclusion

Taken together, the articles contained within this Research Topic provide a comprehensive and clinically relevant overview of the current landscape of surgical management for myelopathies and related conditions. It has been demonstrated that successful treatment is contingent not only on effective decompression and stabilization, but also on precise diagnosis, proper perioperative planning, and proactive complication management. Recent progressions in minimally invasive surgery, intraoperative monitoring, and predictive modelling have collectively resulted in a paradigm shift in clinical practice, thereby facilitating the delivery of more patient-tailored and secure surgical interventions for patients suffering from complex spinal diseases (8). Future research efforts could concentrate on large-scale and multicentric studies integrating an advanced multimodal approach with imaging techniques, intraoperative neurophysiological monitoring, and artificial intelligence-driven predictive models. Such an integration would further refine surgical decision-making processes and optimize personalized treatment strategies for patients suffering from myelopathies.
